# Effective Elimination of Contaminant Antibiotics Using High-Surface-Area Magnetic-Functionalized Graphene Nanocomposites Developed from Plastic Waste

**DOI:** 10.3390/ma13071517

**Published:** 2020-03-26

**Authors:** Noha A. Elessawy, M. H. Gouda, Safaa M. Ali, M. Salerno, M. S. Mohy Eldin

**Affiliations:** 1 Advanced Technology and New Materials Research Institute, City of Scientific Research and Technological Applications (SRTA-City), New Borg El-Arab City, Alexandria 21934, Egypt; 2 Polymer Materials Research Department, Advanced Technology and New Materials Research Institute, City of Scientific Research and Technological Applications (SRTA-City), New Borg El-Arab City, Alexandria 21934, Egypt; marwagouda777@yahoo.com (M.H.G.); mohy108@gmail.com (M.S.M.E.); 3 Nucleic Acid Research Department, Genetic Engineering and Biotechnology Research Institute (GEBRI), City for Scientific Research and Technological Applications (SRTA, City), New Borg El-Arab, Alexandria 21934, Egypt; safaa.mohamedali@yahoo.com; 4 Materials Characterization Facility, Istituto Italiano di Tecnologia, 16163 Genova, Italy; marco.salerno@iit.it

**Keywords:** Garamycin, Ampicillin, magnetic sulfonated graphene, adsorption, ecosystem protection, statistical modeling

## Abstract

The presence of pharmaceutical residues in aquatic environments represents a risk for the equilibrium of the ecosystem and may seriously affect human safety itself in the long term. To address this issue, we have synthesized functional materials based on highly-reduced graphene oxide (HRGO), sulfonated graphene (SG), and magnetic sulfonated graphene (MSG). The method of synthesis adopted is simple and inexpensive and makes use of plastic bottle waste as the raw material. We have tested the fabricated materials for their adsorption efficiency against two model antibiotics in aqueous solutions, namely Garamycin and Ampicillin. Our tests involved the optimization of different experimental parameters of the adsorption process, such as starting antibiotic concentration, amount of adsorbent, and time. Finally, we characterized the effect of the antibiotic adsorption process on common living organisms, namely *Escherichia coli* DH5α (*E. coli* DH5α) bacteria. The results obtained demonstrate the efficiency of the method in addressing the issue of the emergence of antibiotic-resistant bacteria, which will help in preventing changes in the ecosystem.

## 1. Introduction

Pharmaceutical products have been classified as emerging pollutants for the aquatic ecosystems, because they can enter the aquatic environment via different routes, for instance as wastewater from industries and hospitals, as well as the human body excreting to sewage medicines being only partially metabolized [1]. Not only can these be endocrine disruptors, but also generate antibiotic-resistant micro-organisms that will threaten the ecosystem as well as human safety, representing a concern for both.

Aminoglycosides are antibiotics derived from aminated carbohydrates, which are used for the treatment of bacterial infections [2]. They are also used as interactive agents together with other drugs to treat different diseases [3]. Garamycin, viz. the brand name of gentamicin sulfate, is the most common aminoglycoside. This product is used to treat persistent infections caused by gram-negative bacteria [4]. The other commonly used antibiotic is Ampicillin, which is classified as a β-lactam antibiotic and can destroy gram-negative and gram-positive bacteria [5]. However, these antibiotics are not completely biodegradable [6,7,8,9], with the consequence that the residues that remain may induce the development of antibiotic-resistant microorganisms even if the presence of these compounds in the aquatic environment is in low concentration [10].

Many treatment techniques have been studied to eliminate the pharmaceutical compounds from wastewater, including chemical oxidation [11,12], adsorption [12,13,14], membrane techniques [15], and photocatalytic processes [16]. The approach of adsorption is promising, as it shows good efficiency with simple design and low-cost [17]. 

Several studies have demonstrated that graphene-based materials can be used as efficient photocatalysts and adsorbents to remove organic and inorganic pollutants from wastewater [18]. In particular, sulfonated graphene (SG) was synthesized as a derivative of graphene with good dispersibility in water [19] making it a promising material for different applications due to the presence of π–π conjugated structure of graphene. Many researchers have studied the synthesis of graphene with sulfonic groups [19,20], and the reports proved that the process is dependent significantly on three major aspects. The first point involves using the modified Hummers method in graphite oxidative exfoliation to synthesize graphene oxide (GO), followed by the addition of sulfonating agents, such as chlorosulfonic acid [21,22], sulfuric acid [23,24], 4-diazoniobenzenesulfonate [25,26,27], and 2-chloroethanesulfonic acid [28]. On the other hand, SG has been largely used as an adsorbent for many pollutants in aqueous solutions, such as 1-naphthol and naphthalene [29,30], methylene blue (MB), and malachite green (MG) dyes [31], as well as heavy metals [32,33]. One major problem facing SG nanosheets is the ability to retrieve it back from the aqueous solutions after adsorption, thanks to its nanosize and solubility in water, hence, causing secondary pollution in the water. It appears from the literature that decoration of the SG surface with magnetic nanoparticles is beneficial to its adsorption capacity [34] and allows for magnetic materials separation [32]. Usually, the ability of any material for absorption is mostly determined by the number of functional groups available. Thus, the ability of graphene sheets for adsorption could be increased by inserting new functional groups onto the sheets [32,35].

This study aimed to find a solution for purification of antibiotic-contaminated aqueous solutions by using waste materials, according to the “wastes-treat-wastes” idea, to reach both goals of waste management and water treatment. Highly-reduced GO (HRGO), SG, and magnetic SG (MSG) were prepared from polyethylene-terephthalate (PET) resulting from bottle waste [36], as the carbon source. The synthesis proposed here exhibits several advantages, including simple reaction setup and operation, as well as easy and efficient synthesis. The latter point, in particular, seems promising for future scale-up to the industrial level, with possible commercialization of the resulting adsorbent products. Additionally, the recyclable portion of nanoparticle adsorbents is economically significant in the industry. To identify the efficiency of the removal process on the eco-environment, a typical antimicrobial test was studied using *Escherichia coli* DH5α(*E. coli* DH5α).

## 2. Materials and Methods

### 2.1. Starting Materials

Fuming sulfuric acid (H_2_SO_4_), ferrous sulfate (FeSO_4_·7H_2_O), ferric chloride hexahydrate (FeCl_3_.6H_2_O), ammonium hydroxide (NH_4_OH), gentamicin sulfate, and Ampicillin sodium salt were purchased from Sigma Aldrich Supplementary Information Table S1). The PET bottle waste used to prepare HRGO, SG, and MSG nanocomposite was prepared according to the methods described previously [36,37].

### 2.2. Preparation of Graphenic Materials

An appreciable amount of the PET waste was kept in an enclosed autoclave jar and placed in an electric furnace at 800 °C for 1 h. A dark product was produced which was collected and crushed. 

The SG nanosheets were prepared by adding HRGO to concentrated H_2_SO_4_ [24,38], (1 g into 50 mL, Supplementary Information in Figure S1). This was done to insert hydrophilic sulfonic acid groups (SO_3_H) to the HRGO surface. The mixture was ultrasonicated for 30 min and then heated at 150°C under vigorous stirring for 24 h. Afterward, the reaction mixture was cooled down to room temperature (RT), filtered using a vacuum, and washed severally with deionized water to remove excess acid then dried at 80 °C for 12 h. 

Figure S2 shows a MSG nanocomposite synthesized by inverse co-precipitation based on the use of different precursors such as ferric chloride (FeCl_3_·6H_2_O), ferrous sulfate (FeSO_4_·7H_2_O), and ammonium hydroxide (NH_4_OH) as the precipitator [39]. Twenty milliliters of 0.2 M NH_4_OH aqueous solution and 0.5 g of SG nanosheets were added into a 250 mL four-neck bottle beaker under N_2_ atmosphere for 30 min. Then, 1.08 g FeCl_3_·6H_2_O [Fe^2+^] and 0.54 g FeSO_4_·7H_2_O [Fe^3+^] with a stoichiometric ratio of 1:2—resulting in Fe_3_O_4_—were dispersed ultrasonically into 60 mL 1:1 *v/v* water/ethanol solvent. The prepared mixture was poured into a four-neck bottle and set to mechanical stirring for 10 min along with N_2_ bubbling. The produced nanocomposites were separated magnetically, washed with ultrapure water until the resulting solution became neutral, and dried in a vacuum at 80 °C for 12 h.

### 2.3. Characterization of Graphenic Materials

We performed X-ray photoelectron spectroscopy (XPS) with a Phi 5300 ESCA system (Perkin-Elmer) using Mg (Kα) radiation (1253.6 eV). X-ray diffraction (XRD) was performed on a Schimadzu-7000 system, using a Cu Kα radiation beam (λ = 0.154060 nm). Fourier-transformed infrared (FTIR) analysis was carried out through a spectrometer ALFA (Bruker), in a range of 400–4000 cm^−1^. For transmission electron microscopy (TEM) we used a G20 system with EDAX (TECNAI, The Netherlands). Magnetic measurements were carried out at RT with a vibrating sample magnetometer (VSM) model BHV-55 (Riken, Japan). Raman spectroscopy was conducted at RT through a Senterra instrument, (Bruker) with a 514.5 nm excitation wavelength in the range of wave numbers from 40 to 3500 cm^−1^. Materials surface area and pore volume were measured using the Brunauer–Emmett–Teller (BET) technique, with Barret–Joyner–Halenda (BJH) adsorption.

### 2.4. Adsorption Tests

Adsorption tests were carried out using a batch equilibration technique. Aqueous solutions with different antibiotic concentrations (50, 100, 500, and 900 mg L^−1^) were prepared by dilution of the stock of Garamycin or Ampicillin sodium salt (1 g L^−1^) standard solutions. All pH measurements were carried out using a pH meter (Model pHS-25, Shanghai, China) and solutions pH were adjusted using 0.1 M HCl and 0.1 M NaOH. For separation of the solid and the liquid phases after adsorption, for HRGO and SG adsorbent we used centrifugation, whereas for MSG we used a magnet. The concentration of antibiotic in the solution was assessed using UV-visible spectroscopy at a wavelength of 247 and 268 nm for Garamycin and Ampicillin, respectively.

The adsorbed amounts of antibiotic were calculated with these formulas [36,40]

(1) $$q_{t}=\frac{C_{0}-C_{t}}{m}V$$

(2) $$q_{e}=\frac{C_{0}-C_{e}}{m}V$$

where *q_t_* and *q_e_* (mg g^−1^) are the amounts of the antibiotic adsorbed per unit weight of the adsorbent at time t and equilibrium, respectively; *C*_0_, *C_t_*, and *C_e_* (mg L^−1^) are the antibiotic concentrations at the initial time, time t and the equilibrium time, respectively. *V* (L) is the volume of antibiotic solution; m (g) is the mass of adsorbent. Antibiotic removal efficiency (R%) was calculated according to the following equation:

(3) $$R\%=\frac{C_{0}-C_{t}}{C_{0}}\times 100$$

The distribution coefficient (*K_d_*) used for assessing the actual performance of the prepared adsorbents was determined by this equation [41,42]:

*K*_*d*_ = *q*_*e*_/*C*_*e*_ (4)

### 2.5. Efficiency of Garamycin and Ampicillin Adsorption

To check the adsorption of Garamycin and Ampicillin on HRGO, SG, and MSG, we carried out an experiment using E.coli DH5α, which is sensitivity to antibiotic concentrations ≥50 mg L^−1^. The optical density (OD) of culture at 600 nm was measured, after incubation at 37 °C for 24 h. The experiment conditions were: 500 mg L^−1^ antibiotic as starting concentration, 2 mg mL^−1^ of adsorbent dose, pH 5.5. Inoculations with *E. coli* DH5α in the different tubes were identical and done simultaneously in parallel, to allow for comparison of the emerging differences for the different materials.

### 2.6. Adsorption Optimization

We used the response surface methodology models (RSM) to optimize the conditions for removal of the antibiotics. The matrix followed Box–Behnken method [43] with three factors (X1, X2, and X3) and 13 trials [44]. The statistical software "Statistica" was used for data analysis.

### 2.7. Kinetics and Isotherm Modeling

For studies of kinetics, the pH was adjusted to 5.5 and the time of adsorption time was changed in the 5–180 min range. In order to perform the adsorption kinetics, the pseudo first and second order models were used. The adsorption isotherms were tested to validate the antibiotic uptake behavior of the HRGO, SG, and MSG using Langmuir and Freundlich isotherms. 

### 2.8. Adsorption–Desorption Test for MSG

The practical feasibility for MSG was investigated by using regeneration and reuse experiments, whereas 50 mg of MSG after adsorption with Garamycin was dispersed into 0.1 M NaOH in methanol and ultrasonicated for 30 min. Then, the spent-MSG was filtered, washed and dried at 40 °C for 2 h, and subsequently reused. The concentration of Garamycin during this process was measured via UV-visible spectroscopy (Schimadzu, UV-2700i-Double-beam, Double monochromator, Japan). For this study, we carried out five cycles of adsorption and desorption.

## 3. Results and Discussion

### 3.1. Characterization of Graphenic-Based Materials

FTIR spectroscopy was used to investigate the functional groups of the HRGO, SG, and MSG materials. In Figure S3a, the bands at 839 cm^−1^ and 1090 cm^−1^ are associated with the S=O bond [19,45] referring to successfully grafting of the SO_3_H groups onto HRGO, while the band at 1118 cm^−1^ is ascribed to the S-phenyl vibration [46]. Fe–O stretching vibration is noticed at 525 cm^−1^ [32], which is a characteristic peak of MSG sample compared to the HRGO and SG. For all samples the peak at approximately 1720 cm^−1^ is assigned to the C=O bond, while at approximately 3440 cm^−1^ the broad band assigned to the –OH stretching mode of the hydroxyl groups is observed.

The elemental composition and SO_3_H contents of SG and MSG are shown in the Supplementary Information Table S2. 

**Figure 1:**
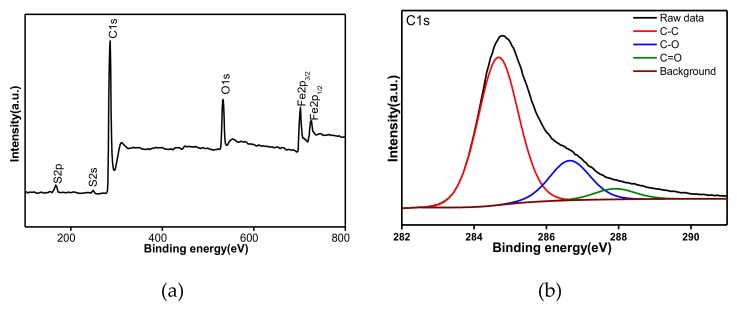

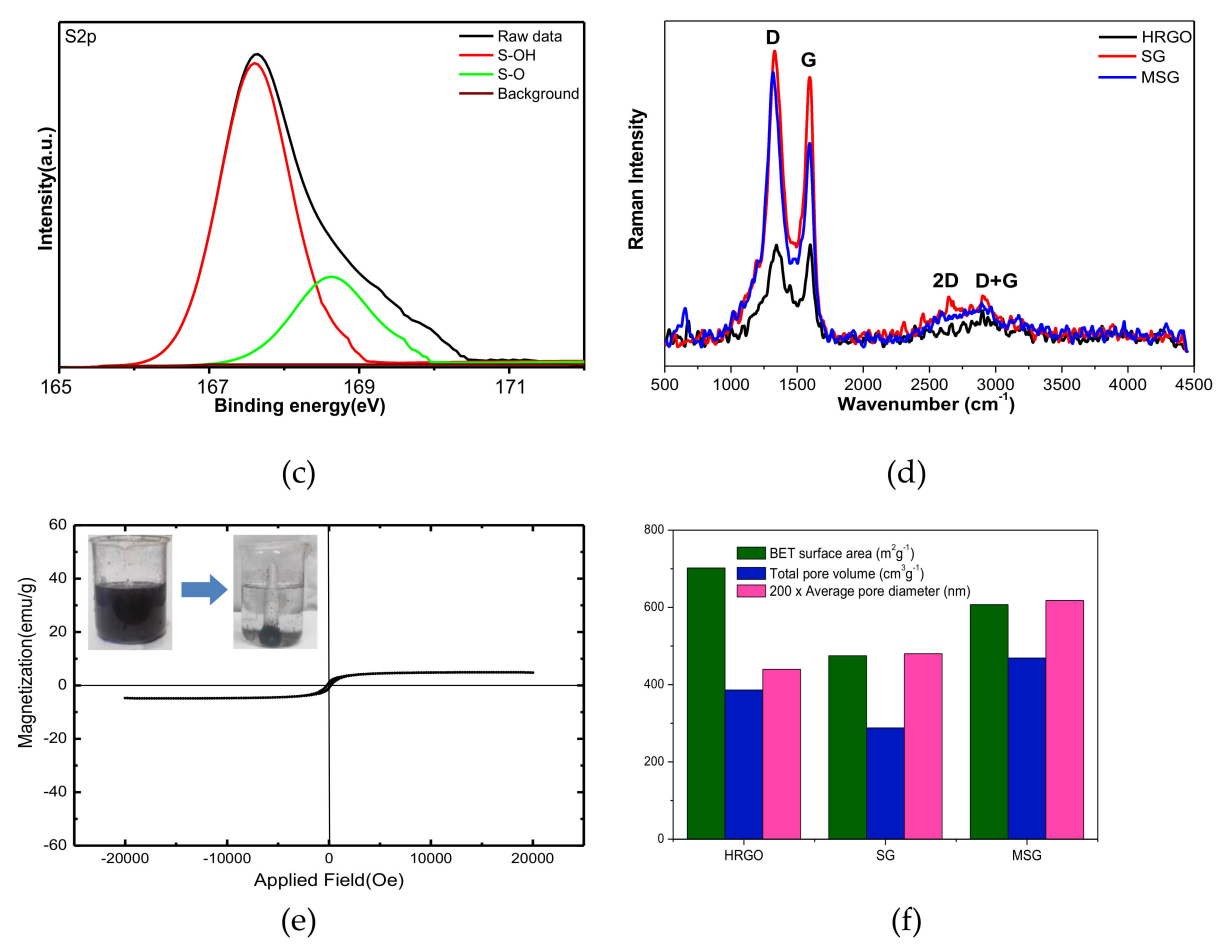
(**a**) X-ray photoelectron spectroscopy (XPS) spectra (**b**) C1s spectra, (**c**) S1s spectra of magnetic sulfonated graphene (MSG) sample, (**d**) Raman patterns, (**e**) Hysteresis loop of the MSG hybrid at room temperature (RT) (The left inset: dispersed and separated particles of MSG by a magnet from the aqueous solution of antibiotic) and (**f**) comparable chart for Brunauer–Emmett–Teller (BET) surface area, average pore diameter and total pore volume of highly-reduced graphene oxide (HRGO), sulfonated graphene (SG), and MSG.

XPS spectra further proved the chemical properties of the MSG sample. In Figure 1a, the absorption peaks of S attributed to S 2p and S 2s appears at a binding energy of 168 and 245 eV, respectively; the peak at 163.9 eV corresponding to sulfide is absent [47], while at binding energies of 286 and 532 eV, the absorption peaks of C and O are noticed. Based on further investigations, as shown in Figure 1b, the high-resolution C 1s XPS spectra show peaks corresponding to C–C, C–O, and C=O. Moreover, as shown in Figure 1c, the high-resolution S 2p spectra show two peaks, the first one at 167.6 eV corresponding to C–S bond energy site between SO_3_H and C, and the second peak at 169.0 eV corresponding to SO_3_H (Table S3). The peak at 163.9 eV corresponding to sulfide is absent. Furthermore, absorption peaks with a binding energy of 712 and 725 eV, attributed to Fe 2p3/2 and Fe 2p1/2, respectively, are observed. As a result, the existence of C, O, S, and Fe elements in the MSG sample is confirmed. 

The powder XRD pattern of HRGO (Figure S3b) shows main peaks at 26° and 44.3° scattering angles, corresponding to the (002) and (100) reflections. On the other hand, for SG a broadened weak peak at approximately 26° is observed, which may attributed to the disordering and irregular interlayer spacing of its graphenic layers [22,28]. However, a slight decrease in the peak at 26° occurs for MSG, due to intercalation of Fe_3_O_4_ nanoparticles between the MSG nanosheets [48,49] at 30.28° (220), 35.78° (311), 53.78° (422), 57.18° (511), and 62.88° (440) for the cubic face-centered structure of Fe_3_O_4_ phase (JCPDS card: 19-0629).

Raman spectra of HRGO, SG, and MSG are shown in Figure 1d. Two main peaks at approximately 1330 and 1590 cm^−1^ correspond to the D and G bands, respectively. The structural disorder level was indicated by the intensity ratio (I_D_/I_G_) of D to G bands [47,50]. I_D_/I_G_ for SG and MSG were evaluated to be 1.1 and 1.3, which are greater than that for HRGO (1.02). This may have been due to the introduction of abundant sulfo-groups to the sp^2^ carbon network in SG, which caused localized sp^3^ defects, in addition to introducing iron species into the MSG. However, the appearance of a broadened weak band ranging from 2500 to 2750 cm^−1^ indicates the highly disordered degree caused by the random arrangement of the graphene sheets [51]. 

Figure 1e shows the magnetic behavior of the synthesized MSG in ambient conditions, which was characterized by VSM. The hysteresis loop in the M–H curve has remanence and the saturation magnetization (Ms) is 4.9 emu g^−1^ with a magnetic field ±20 kOe. This indicates that the synthesized MSG was in a superparamagnetic state. However, the magnetic properties allow for easy separation of the material from aqueous solution under an applied magnetic field. The left inset in Figure 1e shows that the MSG was homogeneously dispersed in aqueous solution and then separated, while the clear solution was removed. 

The specific surface area was investigated by using BET analysis. In Figure 1f we report the results of surface area and pore volume obtained by nitrogen adsorption–desorption isotherms for all the samples (see also Table S4 and Figure S4). According to IUPAC classification, the curves are type-IV isotherms [52,53]. The surface area and the pore volume of SG are slightly lower than that of HRGO, which had a partially damaged porous structure from the sulfonation. On the other hand, the surface area and the pore size of MSG are much higher than those of SG, indicating that the introduction of magnetic nanoparticles avoids aggregation and restacking, leading to a significant increase in the adsorption efficiency.

**Figure 2:**
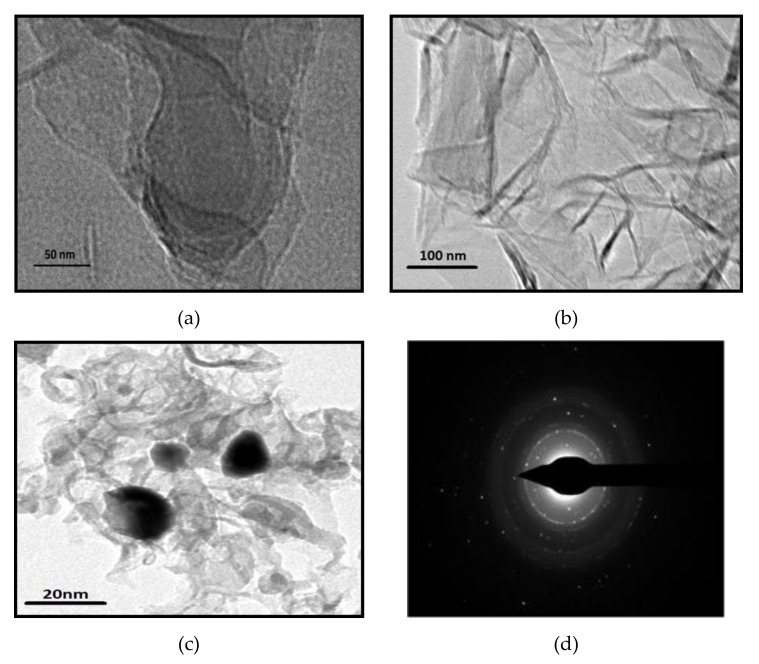
Transmission electron microscopy (TEM) images: (**a**) HRGO, (**b**) SG, (**c**) MSG samples, and (**d**) the selected area electron diffraction (SAED) pattern of MSG sample.

The TEM images in Figure 2a,b, show that HRGO and SG nanosheets exhibit the typical semi-transparent flake morphology resulting after exfoliation, with random aggregation, and overlay and folding of several layers. Differently, in the TEM image of MSG (Figure 2c) the presence of magnetite nanoparticles entrapped in the SG matrix appears clearly. Concurrently, the selected-area electron diffraction (SAED) of MSG (Figure 2d) shows several diffraction rings due to the multilayered graphene assembly, while the sparse, overlaying bright spots are due to the crystalline magnetic nanoparticles.

### 3.2. Effect of Solution pH on the Surface Chemistry

In Figure S5, one can see that as pH increased from 3 to 9, the zeta potential (Supplementary Information) of HRGO, SG, and MSG decreased. This was probably because the surfaces of these materials were highly oxidized, presenting a large number of oxygen functionalities from the sulfonic, carboxylic, and hydroxyl groups, which would be closely related to the active sites. The zeta potential of SG is lower than that of HRGO and MSG at all pH values, which is ascribed to its higher amount of sulfonic groups, which stabilizes the colloidal particles. Therefore SG is water-soluble, which makes its separation from water difficult. 

**Figure 3:**
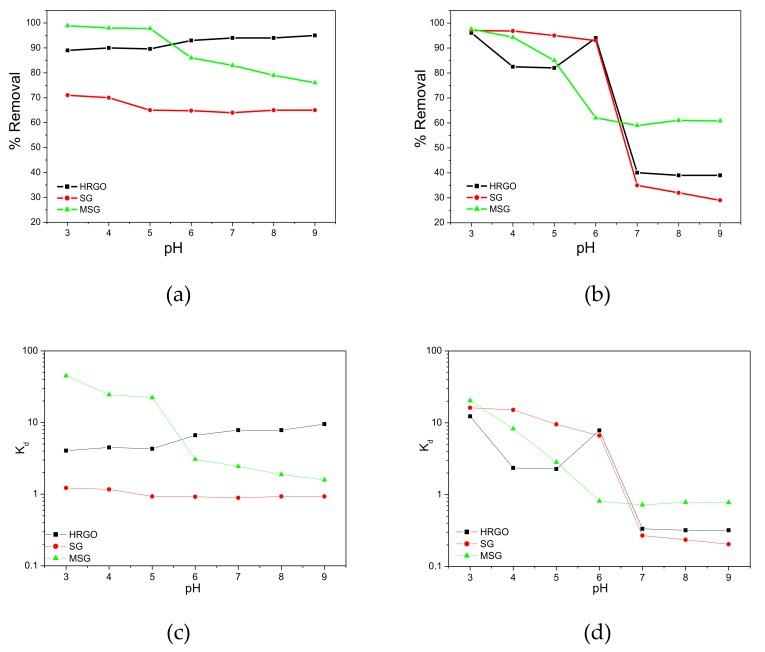
Effect of pH on the removal of: (**a**) Garamycin, (**b**) Ampicillin and effect of pH on K_d_ values for (**c**) Garamycin and (**d**) Ampicillin adsorbed onto HRGO, SG, and MSG [Experimental conditions: C_0_: 500 mg L^−1^; dose of adsorbent: 2 mg mL^−1^; temperature: 25 °C; time: 30 min; volume: 50 mL].

The effect of pH on Garamycin and Ampicillin removal was investigated as shown in Figure 3a,b in the range of 3 to 9, to determine the optimal pH value. For Garamycin, it is noticed that the adsorption capacity is affected by the increasing pH values from 3 up to 9. Many reasons could explain the Garamycin adsorption behavior on the prepared adsorbents relative to the pH values. For example, at low pH, the HRGO, SG, and MSG surfaces are probably surrounded by many hydronium ions, which compete with Garamycin for active sites. In addition, Garamycin solubility can be increased due to the protonation of their amino groups. This could be due to the electron-donating strength being reduced from the protonation of the amino groups, which in turn would decrease the electron donor–acceptor (EDA) interaction between the Garamycin and adsorbents, and result in their uptake on the adsorbents decreasing [54]. In contrast, at a pH below 6, MSG shows a higher tendency of removing cationic Garamycin antibiotic more than HRGO and SG. This could be due to the Fe_3_O_4_ nanoparticles connecting to the SG surface and subjecting the oxidized surface to adsorption of cationic Garamycin antibiotic. The adsorption is due to the electronic density of the MSG surface caused by the Fe_3_O_4_ addition, and would prompt low bonding energy with Garamycin molecules. Conversely, at high pH values, the assembly of –(OH) in the solution would react with hydrogen dissociation from amino groups, and the Garamycin surface would be less positively charged, causing the uptake on the adsorbent surface to decrease. Therefore, a pH of 5.5 was selected as the optimum pH for removing Garamycin in the other experiments.

For Ampicillin, which has amphoteric properties (Figure S6), by increasing the pH of the solution, the adsorbent surface charge decreases due to deprotonation of oxygen and more –COOH groups changing into (COO)^−^, so the repulsion between Ampicillin and the adsorbents increases. However, in an acidic solution, an electrostatic interaction occurs between the positively charged Ampicillin sodium molecules and the adsorbent surface. A relative repulsion could occur with the MSG at a pH of 6, which would be possible due to the Fe_3_O_4_ surface at that pH having a net positive charge (the point of zero charge of Fe_3_O_4_ is between 6 and 6.4) [55]. Therefore, MSG can adsorb Garamycin more efficiently, while SG can adsorb Ampicillin efficiently in a pH solution of adsorbate and adsorbent, as shown in the Supplementary Information Table S5. Figure 3c,d shows the distribution coefficient (K_d_) over a pH range of 3 to 9, and its calculated values at optimum pH in each case are represented in the Supplementary Information Table S6. It is noted that the distribution coefficient is sensitively affected by the changes in solution pH, and consequently affects the adsorption capabilities of the adsorbents.

### 3.3. Speculations on the Adsorption Mechanism

According to literature, the main adsorption interactions of antibiotics on graphene-based nanomaterial surfaces are π–π interactions, H-bonding, hydrophobic interactions, cation–π bonding, electrostatic forces, and π–π stacking [56,57]. The most important properties of adsorptive materials that play an important role in the adsorption process are internal properties and surface morphology. However, the prepared materials have heterogeneous surface due to the coexistence of several different groups, which might express different mechanisms of adsorption.

It has been suggested that the adsorption of Garamycin and Ampicillin on HRGO, SG, and MSG depends on two groups of reasons: the first includes functional groups, surface area, pore size, and uniformity in the adsorbents; the second includes a number of functional groups and aromatic rings in the antibiotic molecule, which induces π–π EDA interactions with the adsorbent [56]. Therefore, it is concluded that the more functional groups and aromatic rings the antibiotics have, the faster is the adsorption rate on the adsorbent surface. The prepared HRGO, SG, and MSG typically have a high surface area that, if exposed to the solution phase, could provide surface sites for rapid and extensive adsorption of antibiotic molecules. However, the adsorption capacity ranking appearing as MSG > HRGO > SG is due to the functional groups contained at the sp^3^-hybridized edge of graphene, as well as the surface area due to total pore volume and average pore diameter. Besides, the strong adsorptive interaction is mainly due to the electron-donating effect of the amino group, which causes a strong EDA interaction between Garamycin and the π-electron depleted regions on the adsorbents’ surface [58]. In contrast, the dominant mechanisms of cationic Ampicillin molecule adsorption on the functionalized graphene adsorbents at low pH are also π–π interaction and electrostatic attraction. The adsorption capacity of Ampicillin on HRGO is due to the –OH and –COOH groups in the plane, and the π–π interaction between HRGO and Ampicillin molecules. When functionalized with a –SO_3_H group, the effective sites of HRGO for adsorbing Ampicillin molecules increase, due to the sulfonic group attracting strongly the positively charged molecules. Hence, the absorbability of MSG is weaker than that of SG in a pH solution greater than 6.

### 3.4. Efficiency of Garamycin and Ampicillin Adsorption

**Figure 4:**
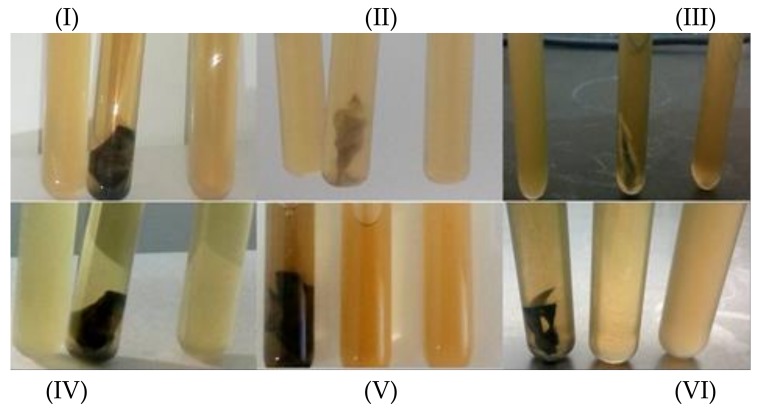
Confirmation test of the efficiency of the adsorption process on E.coli growth (at initial concentration 500 mg L^−1^, adsorbent dose 2 mg mL^−1^, and pH 5.5) represented by three tubes, containing E.coli culture, *E.coli* culture with filtrated adsorbent loaded with antibiotics, and E.coli culture with filtrate solution, respectively. (**I**) Garamycin/MSG, (**II**) Garamycin/SG, (**III**) Garamycin/HRGO, (**IV**) Ampicillin/ SG, (**V**) Ampicillin/ MSG, and (**VI**) Ampicillin/ HRGO.

As shown in Figure 4, the experiment presented three different tubes, containing *E. coli* culture, *E. coli* culture with filtrated adsorbent loaded with antibiotics, and *E. coli* culture with filtrate solution, respectively. For image I, the left tube was the control sample (*E. coli* DH5α), which gives high growth (high OD), the middle tube contained MSG which adsorbed Garamycin and showed no growth of bacteria, and the right tube contained filtrated water causing high bacterial growth (there is no difference between it and the control). The result reveled that MSG adsorbed all of Garamycin. In Image II, the left tube is a control sample (*E. coli* DH5α) which resulted in high growth, the middle tube contains SG, which adsorbed the amount of Garamycin and bacteria growth was moderate, and the right tube has filtrated water, which caused moderate bacterial growth and means the presence of Garamycin after the adsorption process. Image III shows the left tube, which is the control sample (*E. coli* DH5α ) with high growth, the middle tube contains HRGO, which adsorbed Garamycin showing no bacteria growth, and the right tube contains filtered water and has low bacterial growth, which means there is a small amount of Garamycin left in the water. Image IV shows the control sample (*E. coli* DH5α) in the left tube with high growth, the middle tube is SG, with adsorbed Ampicillin and no bacteria growth, and the right tube with filtered water that caused low bacterial growth, which means there is a small amount of Ampicillin left in the water. In image V, the left tube is MSG loaded with Ampicillin and its bacteria growth was moderate, the middle tube is filtered water which caused moderate bacterial growth (there is still Ampicillin in the water), and the right tube is the control sample (*E. coli* DH5α) with high growth. In image VI, the left tube was HRGO-adsorbed Ampicillin and the bacteria growth was moderate, the middle tube was filtered water which caused moderate bacterial growth, and the right tube is the control antibiotics such sample (*E. coli* DH5α) with high growth. It was observed that the prepared adsorbents were able to remove as Garamycin and Ampicillin, which are present in wastewater. This could help overcome the problem of the appearance of antibiotic-resistant microbes, and thus prevent changes in the ecosystem.

### 3.5. Optimization of Adsorption 

The adsorption processes were analyzed for optimization using a Box–Behnken design [43] with three variables, namely time, starting antibiotic concentration, and amount of adsorbent (see Table S7 for Garamycin on MSG and Ampicillin on SG). We could assess a statistical relation between the variables and the responses (see Tables S8 and S9), which is described using a quadratic model as follows:

Y_gara_ = 98.7 + 0.65X_1_ − 3.2625X_2_ + 1.5375X_3_ − 0.525X_1_^2^ − 3.05 X_2_^2^ − 0.8X_3_^2^ + 0.175 X_1_X_2_ − 0.275X_1_X_3_ + 0.5X_2_X_3_ (5)

Y_amp_ = 93.1 + 2.5875X_1_ − 5.3125X_2_ + 2.65X_3_ − 1.15X_1_^2^ + 0.9X_2_^2^ − 2.475X_3_^2^ + 1.45X_1_X_2_ + 1.625X_1_X_3_ + 1.825X_2_X_3_ (6)

where Y is the response (antibiotic removal yield) and X_1_, X_2_, and X_3_ are the above-mentioned factors of time, starting antibiotic concentration, and amount of adsorbent.

**Figure 5:**
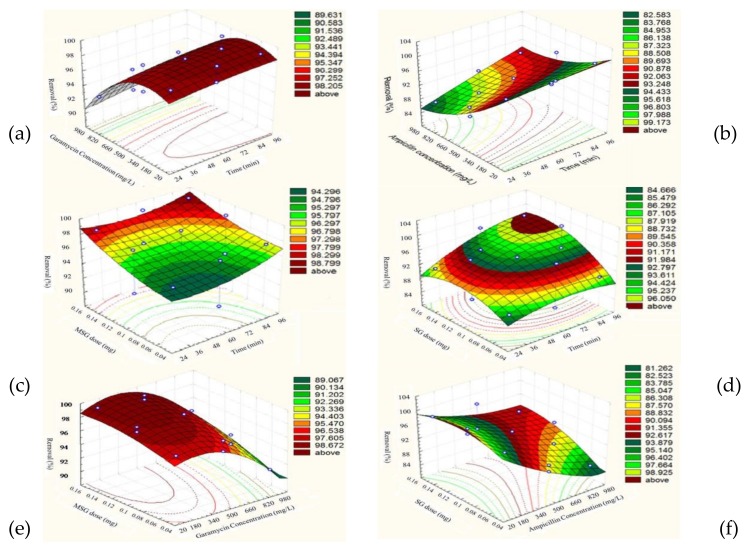
(**a**,**c**,**e**) represent Surface plots of response for removal efficiency (%) of Garamycin on MSG, while plots (**b**,**d**,**f**) represent Surface plots of response for removal efficiency (%) of Ampicillin on SG.

Figure 5 shows that, to remove Garamycin completely according to the above equations, the best conditions were 69 min, 314.84 mg L^−1^ of Garamycin, and 0.13786 mg of MSG, at a pH of 5.5 and 25 °C. However, at contact time 83.9 min, 100 mg L^−1^ of Ampicillin concentration and 0.121419 mg of SG, the maximum removal efficiency of approximately 99.94% of Ampicillin on SG was achieved at a solution pH of 5.5 and 25 °C.

### 3.6. Adsorption Kinetic Models

From Table 1, by comparing the experimental capacity of antibiotic adsorption capacities with the calculated one, we observed a vast difference between the obtained values (Figure S7). Hence, the pseudo-first-order reaction kinetic model (Figure S8 (a) and (c)) was inadequate to depict adsorption of Garamycin and Ampicillin on MSG and SG, respectively. Whilst for the pseudo-second-order kinetic model (Figure S8 (b) and (d)), it is dependent on the initial concentration of adsorbate adsorbed on the surface of the adsorbent and the adsorbed concentration at equilibrium [40] it was observed that the q_e,_ calculated values corresponded to the experimental q_e_ values (q_e_, exp), and the obtained correlation coefficients (R^2^) were higher than those for the pseudo-first-order. Therefore, the adsorption processes follow closely the pseudo-second-order model, which explains the controlling rate step as chemisorption and the rate of adsorption for both antibiotics is dependent on the accessibility of available adsorption sites on the surface of the adsorbent materials. Also, as the concentration of the antibiotic increased, the k_2_ values decreased, this may also be attributed to an increase in competition for adsorption sites compared to the low antibiotic concentrations [56]. 

### 3.7. Adsorption Isotherms

Adapting Langmuir and Freundlich adsorption isotherm models [40,59] as illustrated in Table 1 and Figure S9, the Langmuir model best represented the adsorption data with effective adsorption behavior at high temperatures. However, in the Freundlich model, the 1/n_F_ values, which describe the adsorption intensity or surface heterogeneity, varied in the 0–1 range, which means a vast heterogeneity as the values approached zero. Thus, the adsorption of both antibiotics was effective using Langmuir and Freundlich isotherm models.

Various adsorbent materials were tested to remove Garamycin and Ampicillin from water as illustrated in Table 2.

### 3.8. Adsorption–Desorption Test for MSG

**Figure 6:**
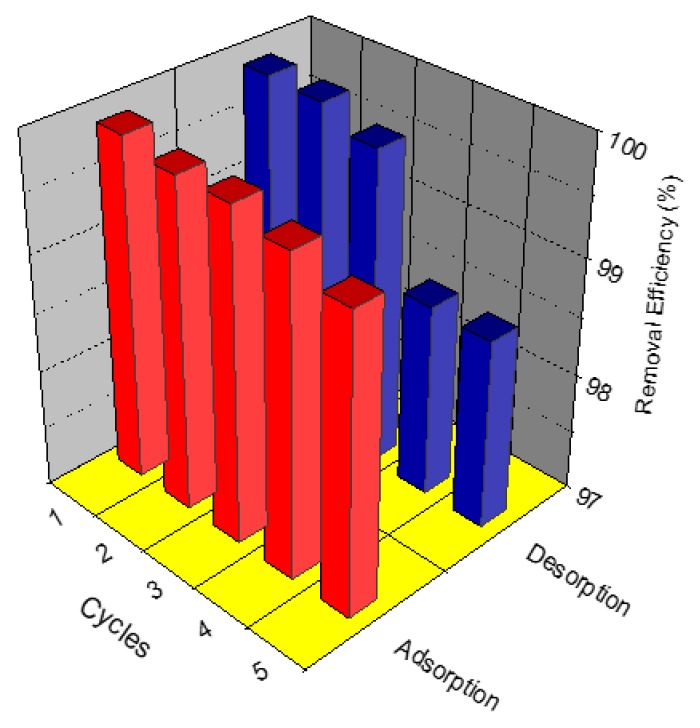
Adsorption–desorption cycles of Garamycin onto MSG. (Conditions: C_0_, 20 mg L^−1^; adsorbent dose, 2 mg mL^−1^; and pH, 5.5).

The ability to regenerate MSG is a key point to evaluate its economic importance. In Figure 6, one can see that the removal efficiency was high even after five cycles and after each desorption process, the variations in the removal percentages were inconspicuous. This makes MSG a potential adsorbent in wastewater treatment.

## 4. Conclusions

The method of post-modification requires the complex and critical process of the reduction of GO followed by sulfonation. In this work, SG contained SO_3_H functional groups obtained directly by means of a simple process, as detailed in the report. This process is more efficient, inexpensive, and simple in preparing SG and MSG than any previously reported method. The results of the present experiments show that, the adsorbent’s porosity, surface area, and functional groups present on the adsorbent and antibiotics are significant operating parameters affecting both the efficiency and the rate of adsorption. In conclusion, the prepared graphene-based materials exhibit superior properties for adsorption of Garamycin and Ampicillin in both high and low environmental concentrations, and thus can help to maintain the ecosystem and preserve all organisms from undesired change.

## Figures and Tables

**Table 1:** Parameters and determination coefficients of the kinetic and isotherm models for Garamycin and Ampicillin adsorption on prepared MSG and SG.

**Kinetic Models Parameters**	**Garamycin Starting Concentration (mg L^−1^) Adsorbed on MSG**			**Ampicillin Starting Concentration (mg L^−1^) Adsorbed on SG**		
	**100**	**500**	**900**	**100**	**500**	**900**
q_e,exp_ (mg/g) *	50	242	411	50	226	378
Pseudo-1^st^-order						
q_e’cal_(mg/g)	6.6	51.9	139.7	3.4	28.6	224.8
k_1_(min^−1^)	0.05	0.04	0.04	0.03	0.04	0.04
R^2^	0.991	0.984	0.982	0.993	0.961	0.984
Pseudo-2^nd^-order						
q_e,cal_(mg/g)	50	245	414	50.2	235	383
k_2_(min^−1^)	0.001	0.002	0.001	0.001	0.003	0.002
R^2^	0.999	0.998	0.997	0.998	0.994	0.993
	**Garamycin Adsorption on MSG**			**Ampicillin Adsorption on SG**		
**Temperature (°C)**	**25 °C**	**35 °C**		**25 °C**	**35 °C**	
Langmuir isotherm						
q_m_ (mg/g)	456.6	473.9		500	384.6	
k_L_(L/mg)	0. 113	0.547		0.04	0.65	
R^2^	0.998	0.997		0.998	0.995	
R_L_	0.01	0.002		0.027	0.002	
Freundlich isotherm						
K_F_(mg/g)	61.6	88.6		79.04	123.3	
1/n_F_	0.39	0.35		0.3	0.2	
R^2^	0.999	0.999		0.998	0.996	

**Table 2:** A review of the performance of some adsorbent materials used to remove Garamycin and Ampicillin from water.

Adsorbent Nanomaterials	Adsorbate	Optimum Adsorption Condition (Temperature °C, pH)	Adsorbate Initial Concentration (mg L^−1^)	Maximum Adsorption Capacity (mg g^−1^)	Reference
Silicas (SILs)	Garamycin	------	478	49.42	[60]
silica matrices (SBA-15-NH_2_)	Ampicillin	25, 7.4	------	333	[59]
Granular Activated Carbon (GAC)	Ampicillin	25, 6	750	12.7	[61]
Carbon Materials (CM2) Nitrogen Treated Carbon Materials (CM1)	Ampicillin	25, 7	1048 1048	206 178	[62]
HRGO	Garamycin	25, 6	500	232.5	This work
	Ampicillin	25, 6	500	235	This work
SG	Garamycin	25, 5.5	500	170	This work
	Ampicillin	25, 5.5	500	233.75	This work
MSG	Garamycin	25, 5.5	500	240	This work
	Ampicillin	25, 5.5	500	183.5	This work

## Supplementary Materials

The following are available online at https://www.mdpi.com/1996-1944/13/7/1517/s1, **Figure S1**: Proposed scheme for the formation of SG and MSG. **Figure S2**: Scheme of the preparation of MSG nanocomposite. **Figure S3**: (a) FTIR pattern and (b) XRD pattern of HRGO, SG, and MSG samples. **Figure S4**: N2 adsorption–desorption isotherms for the prepared HRGO, SG, and MSG at 77 K (inset: comparable chart for BET surface area, total pore volume, and average pore diameter of HRGO, SG, and MSG). **Figure S5**: Zeta potential of HRGO, SG, and MSG. **Figure S6**: Dissociation of Garamycin and Ampicillin at different pH. **Figure S7**: Effect of contact time on the amounts of Garamycin and Ampicillin adsorbed per unit weight of MSG and SG, respectively (C0: 100 mg L−1; dose of adsorbent: 2 mg/mL; temperature: 25 °C). **Figure S8**: Pseudo-first-order and pseudo-second-order kinetic models for the adsorption of Garamycin on MSG (a) and (b), respectively, and of Ampicillin on SG (c) and (d), respectively (Conditions: dose of adsorbent: 2 mg/mL, temperature: 25 °C). **Figure S9**: Langmuir and Freundlich isotherm models for the adsorption of Garamycin on MSG (a) and (b), respectively, and of Ampicillin on SG (c) and (d), respectively (Adsorbent dose: 2 mg/mL). **Table S1**: Representative drug with their, antibiotics classification, IUPAC nomenclature, chemical structure, molecular formula and molecular weight. **Table S2**: The elemental composition and SO3H contents of SG and MSG. **Table S3**: Analysis of the main elements in the XPS survey spectra of MSG (At.% = atom%). **Table S4**: The texture features (surface area, pore volume, and size) for G, SG and MSG. **Table S5**: pH values of the different solutions. **Table S6**: Performance figures of the prepared nanomaterials used for Garamycin and Ampicillin removal. **Table S7**: Central composite matrix of experimental and predicted values for Garamycin removal (%) using prepared MSG, and Ampicillin removal (%) using prepared SG, at solutions pH 5.5. **Table S8**: ANOVA analysis of the selected factors on the adsorption efficiency of MSG for Garamycin in aqueous solution. **Table S9**: ANOVA analysis of the selected factors on the adsorption efficiency of SG for ampicillin in aqueous solution.
