# Supplementary material for: Effective Elimination of Contaminant Antibiotics Using High-Surface-Area Magnetic-Functionalized Graphene Nanocomposites Developed from Plastic Waste
Source: Materials (Basel). 2020 Mar 26;13(7):1517. doi: 10.3390/ma13071517 (PMC7177265; doi:10.3390/ma13071517)
Supplement: Supplementary file 1 [file materials-13-01517-s001.pdf]

Supplementary Materials

Effective Elimination of Contaminant Antibiotics Using High-Surface-Area Magnetic-Functionalized Graphene Nanocomposites Developed from Plastic Waste

Noha A. Elessawy[1,*], M. H. Gouda[2], Safaa M. Ali[3], M. Salerno[4] and M. S. Mohy Eldin[2]

1 Advanced Technology and New Materials Research Institute, City of Scientific Research and Technological Applications (SRTA-City), New Borg El-Arab City 21934, Alexandria, Egypt.

2 Polymer Materials Research Department, Advanced Technology and New Materials Research Institute, City of Scientific Research and Technological Applications (SRTA-City), New Borg El-Arab City 21934, Alexandria, Egypt; marwagouda777@yahoo.com (M.H.G.), mohy108@gmail.com (M.E.)

3 Nucleic Acid Research Department, Genetic Engineering and Biotechnology Research Institute (GEBRI), City for Scientific Research and Technological Applications (SRTA, City), New Borg El-Arab, 21934 Alexandria, Egypt; safaa.mohamedali@yahoo.com.

4 Materials Characterization Facility, Istituto Italiano di Tecnologia, 16163 Genova, Italy; marco.salerno@iit.it.

* Correspondence: nony_essawy@yahoo.com

Received: 13 February 2020; Accepted: 19 March 2020; Published: date

Characterization of HRGO and Its Derivatives

An X-ray Photoelectron Spectroscopy (XPS) Phi 5300 ESCA system (Perkin-Elmer, U.S.A) with Mg (Kα) radiation (X-ray energy 1253.6 eV) was used. X-ray Diffraction (XRD) data (Schimadzu-7000, U.S.A.) was collected with a CuKα radiation beam (λ = 0.154060 nm). Fourier Transform Infrared (FTIR) analysis was conducted using a Bruker ALFA FTIR spectrometer with a range from 400 to 4000 cm^{-1}. A Transmission Electron Microscope (TEM) (TECNAI G20, Netherland with EDX) was also used. A Vibrating Sample Magnetometer (VSM, BHV-55, Riken, Japan) was used for magnetic measurements at room temperature. Raman spectroscopy was conducted at room temperature using a Senterra Raman spectrometer, (Bruker- Germany) with a 514.5 nm excitation wavelength in the range of wave numbers from 40 to 3500 cm^{-1}. The Brunauer–Emmett–Teller (BET) surface area and total pore volume were measured using Barret–Joyner–Halenda (BJH) adsorption methods.

Acid Groups' Content and Zeta Potential Measurement

The content of acid groups was determined by acid-base titration for SG and MSG, where 0.5 g of SG or MSG was sonicated in 50 mL of 1 M NaCl for 24 h. The suspension was titrated slowly with 0.01 M NaOH solution to reach the neutral point (pH 7) and was monitored using phenolphthalein as an indicator.
The equivalent weight (EW) was calculated using:

$$EW = \frac{W}{V_{NaOH}\, C_{NaOH}} \quad (1)$$

where V_{NaOH} and C_{NaOH} are the volume and concentration of NaOH solution used in the titration and W is the dry weight of SG or MSG samples. The EW for SG and MSG was found to be 0.7 and 0.37 g mol^{-1}.

For Zeta potential measurements, 0.05 g of HRGO, SG, or MSG was added into 10 mL of 1M NaCl solutions and the suspensions were sonicated until fully dispersed. The pH of the suspensions was adjusted from pH 3 to 9 using 0.1 M NaOH or HCl, and the Zeta potential was then determined using a Malvern Nanosizer Zeta potential.

Efficiency Verification of Garamycin and Ampicillin Adsorption Processes

To verify the efficiency of Garamycin and Ampicillin adsorption processes on HRGO, SG, and MSG, an experiment using E. coli DH5α, which has sensitivity to antibiotic concentration ≥50 ppm, was conducted. The experiment measured the optical density (OD) of culture absorbance at 600 nm after 24 h incubation at 37 ˚C. The experiment conditions were 500 mg L^{-1} antibiotic as initial concentration, a dose of adsorbent of 2 mg mL^{-1}, solution pH was adjusted at 5.5, and inoculated with E. coli DH5α were identical and used at the same time in the tubes to evaluate the material difference.

Kinetics of adsorption process

Kinetics study was important as it describes the uptake rate of adsorbate. The rate and mechanism of the antibiotics adsorption process on adsorbent could be elucidated based on kinetic studies. In order to elucidate the adsorption kinetics, the pseudo-first-order and pseudo second-order models were applied.

$$q_t = q_e(1 - e^{-k_1 t}) \tag{2}$$

$$q_t = \frac{k_2 {q_e}^2 t}{1 + k_2 q_e t} \times 100 \tag{3}$$

where k_1 and k_2 are pseudo-first-order and pseudo-second-order adsorption rate constants, respectively.

The pseudo-first-order kinetic model is more suitable for low concentration of solute. It can be written in the following form:

$$\ln(q_e - q_t) = \ln q_e - k_1 t \tag{4}$$

where $q_e$is the amount of dye adsorbed at saturationper gram of adsorbent (mg g^{-1}), q_t is the amount of dye adsorbed at time t per gram of adsorbent (mg g^{-1}), and k_1 (min^{-1}) is the rate constant of the pseudo first-order adsorption. While, the pseudo-second-order kinetic model is dependent on the solute amount adsorbed on the surface of adsorbent and the adsorbed amount at equilibrium.

Adsorption Isotherms

The antibiotics sorption capacity of the prepared material at different initial concentrations at equilibrium can be illustrated by the adsorption isotherms. Adsorption isotherms describe how the adsorbate interacts with adsorbents and give a thorough understanding of the nature of interaction. Several isotherm equations have been developed and employed for such analysis and the two important isotherms were applied.

Langmuir Isotherm Model

Langmuir's isotherm was used for monolayer adsorption on a surface containing a finite number of identified sites with negligible interaction between adsorbed molecules and assumes uniform energies of adsorption on the surface. In addition the maximum adsorption depends on the saturation level of monolayer. The Langmuir isotherm is represented by the following linear equation:

$$\frac{q_e}{C_e} = \frac{1}{K_L q_m} + \frac{C_e}{q_m} \tag{5}$$

where q_e is the solid-phase antibiotic concentration in equilibrium with the liquid-phase concentration C_e expressed in mole L^{-1}, q_m is the maximum monolayer adsorption capacity (mg g^{-1}), and K_L is an equilibrium constant (L mol^{-1}). A straight line with slope of $1/q_m$ and intercept of $1/K_L q_m$ is obtained when C_e/q_e is plotted against C_e. The separation factor (R_L) is a dimensionless constant which is an essential characteristic of the Langmuir model. The equation of R_L is expressed as:

$$\frac{q_e}{C_e} = \frac{1}{K_L q_m} + \frac{C_e}{q_m} \tag{6}$$

$$R_L = \frac{1}{1 + K_L C_0} \tag{7}$$

where C_o (mg L^{-1}) is the highest studied initial antibiotic concentration, (Co = 900 mg L^{-1}).R_L indicates if the isotherm is unfavorable when $R_L>1$, linear at $R_L=1$, favorable at $0<R_L<1$, or irreversible at $R_L=0$.

Freundlich Isotherm Model

Adsorbents that follow the Freundlich isotherm equation are assumed to have a heterogeneous surface consisting of sites with different adsorption potentials, and each type of site is assumed to adsorb molecules, as in the Langmuir equation:

$$lnq_e = lnK_f + \frac{1}{n\ lnC_e} \tag{8}$$

where K_f is constant (function of energy of adsorption and temperature) and n is a constant related to adsorption intensity, by plotting lnq_e versus ln C_e which gave a straight line with slope of 1/n and intercept of lnK_f. The magnitude of the "n" shows an indication of the favorability of adsorption.

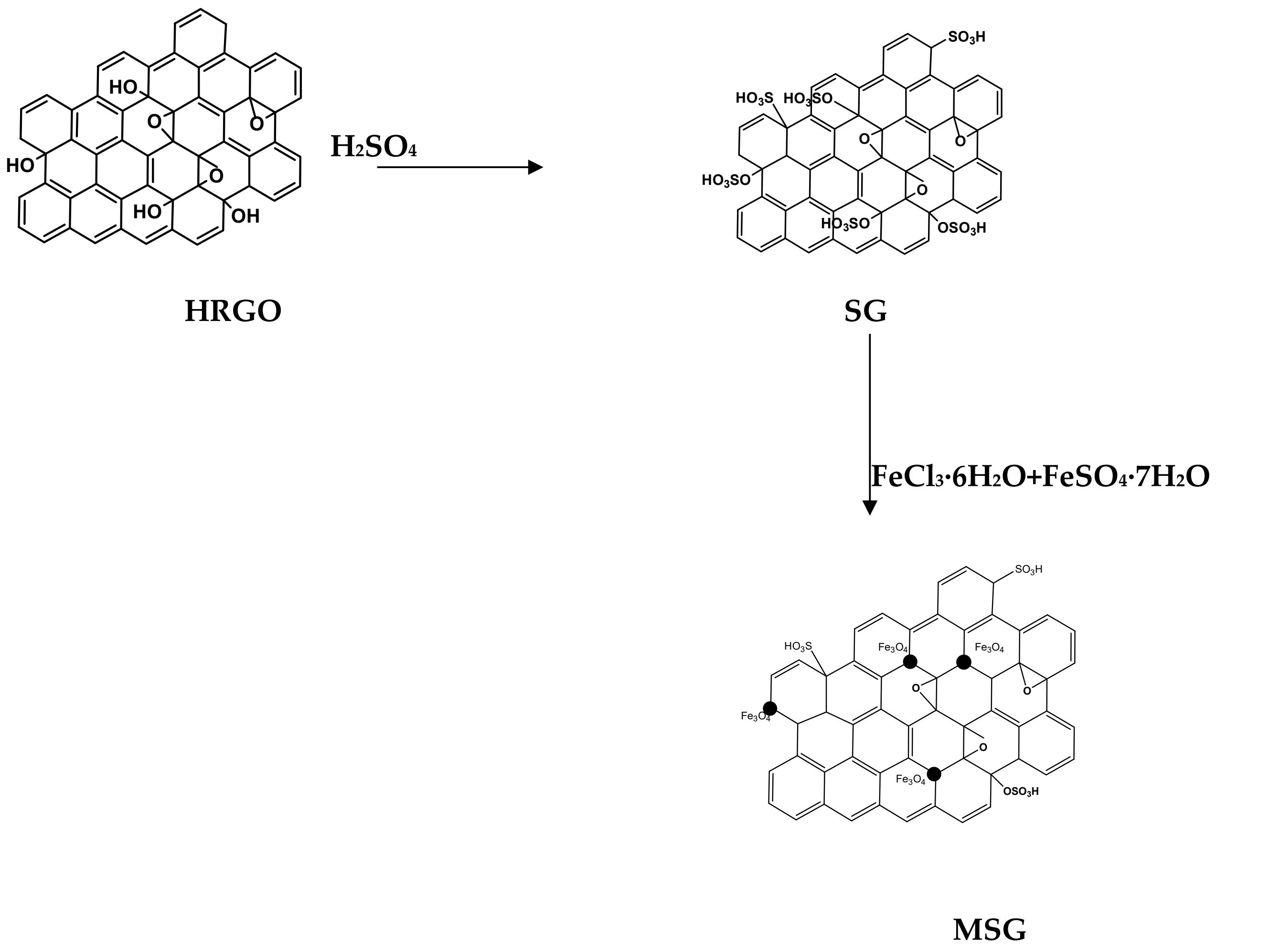

Figure S1. The proposed scheme for the formation of SG and MSG.

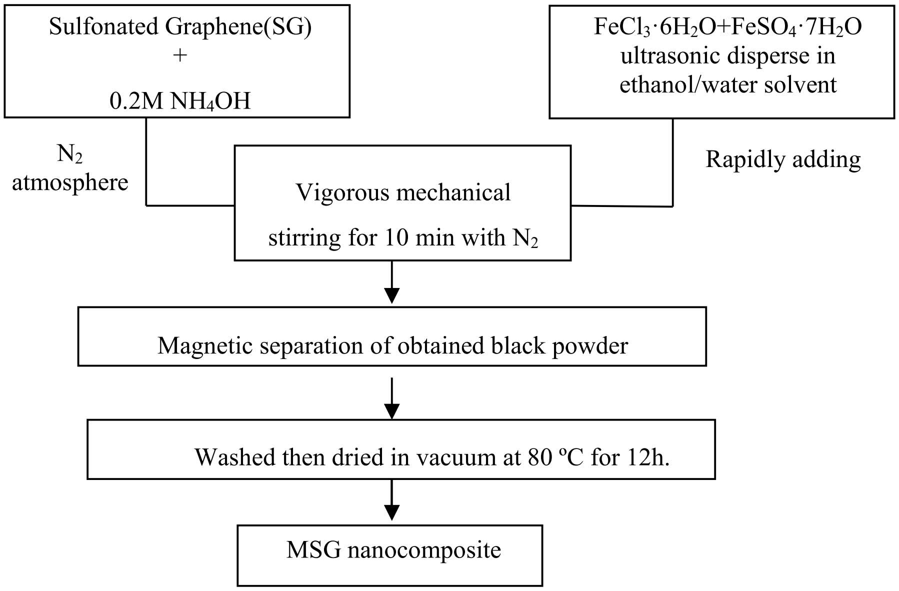

Figure S2. The scheme of the preparation of MSG nanocomposite.

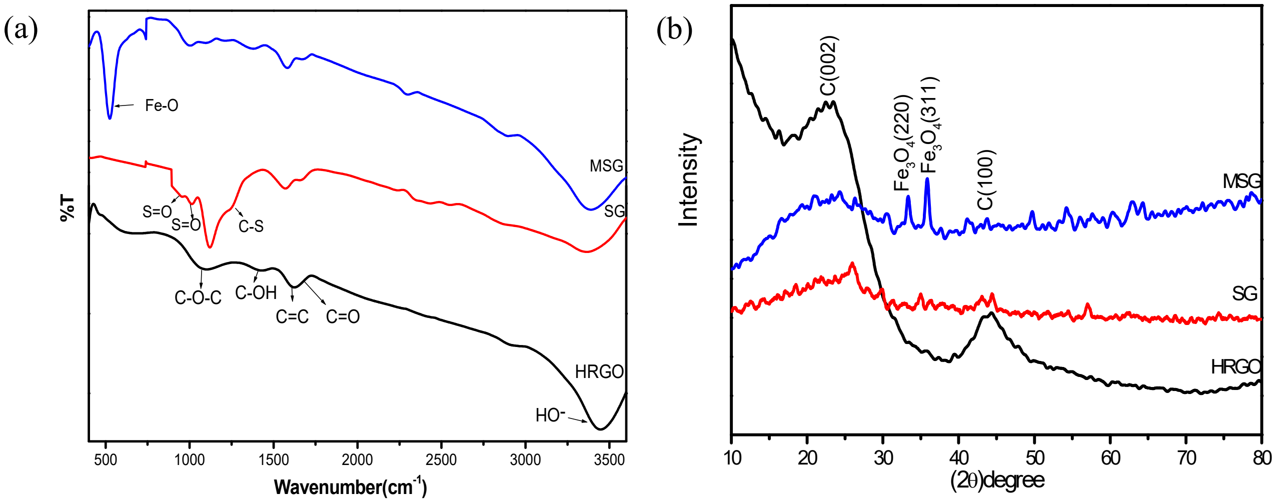

Figure S3. (**a**) FTIR pattern and (**b**) XRD patternof HRGO, SG, and MSG samples.

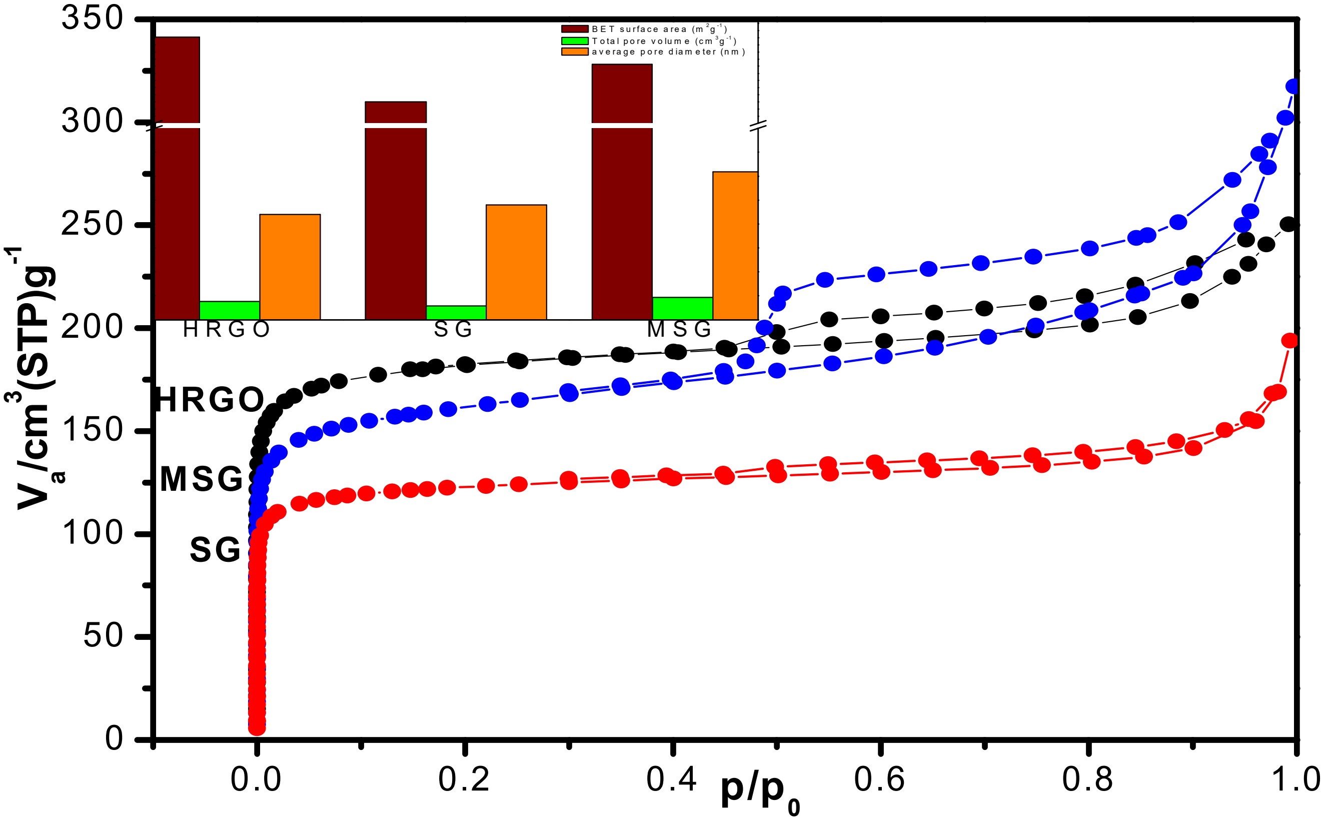

Figure S4. N_2 adsorption–desorption isotherms for the prepared HRGO, SG, and MSG at 77 K (inset: comparable chart for BET surface area, total pore volume, and average pore diameter of HRGO, SG, and MSG).

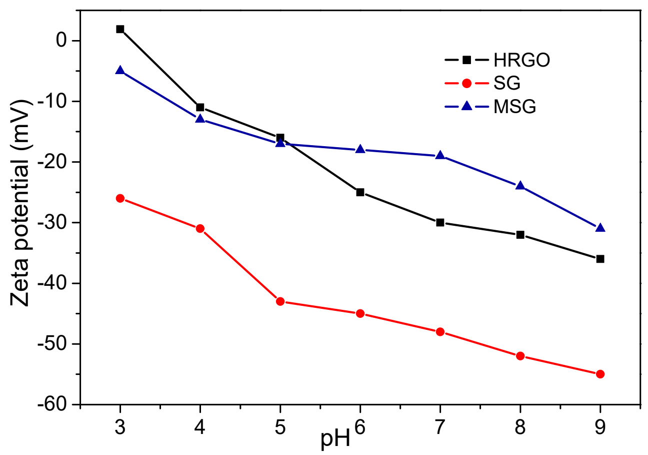

Figure S5. Zeta potential of HRGO, SG, and MSG.

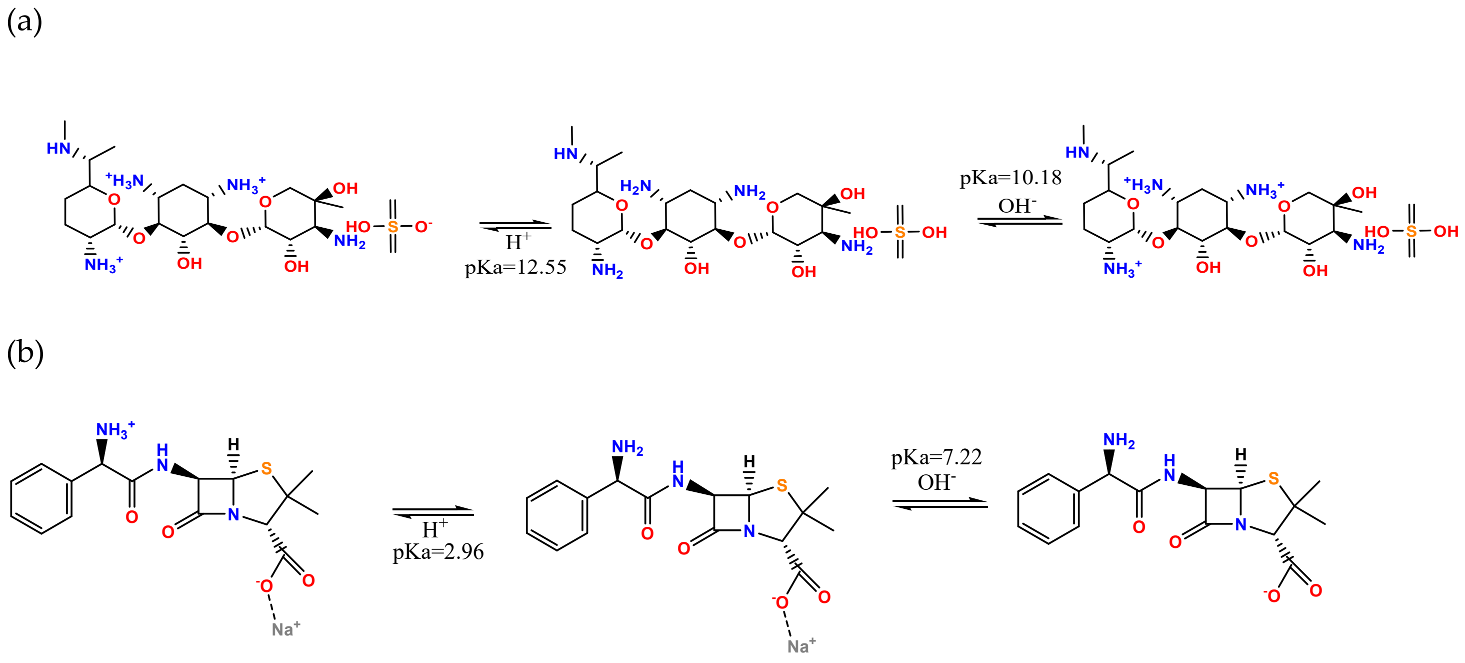

Figure S6. Dissociation of Garamycin and Ampicillin at different pHs.

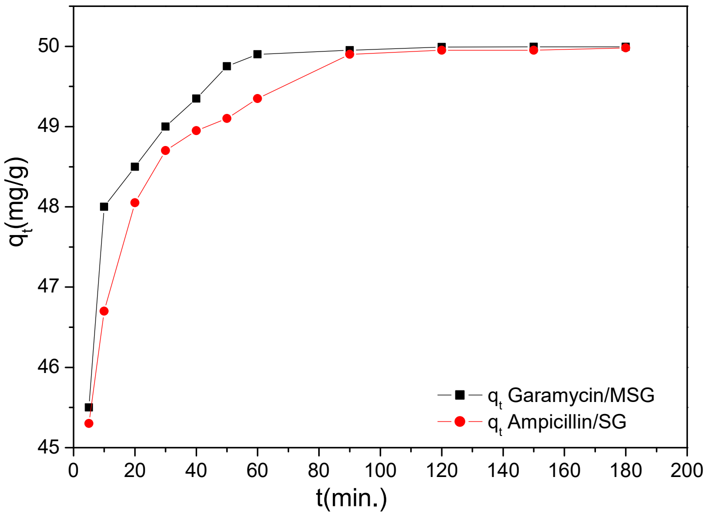

Figure S7. Effect of contact time on the amounts of Garamycin and Ampicillin adsorbed per unit weight of MSG and SG respectively [C_0: 100 mg L^{-1}; Dose of adsorbent: 2 mg/ml; Temperature: 25 °C]

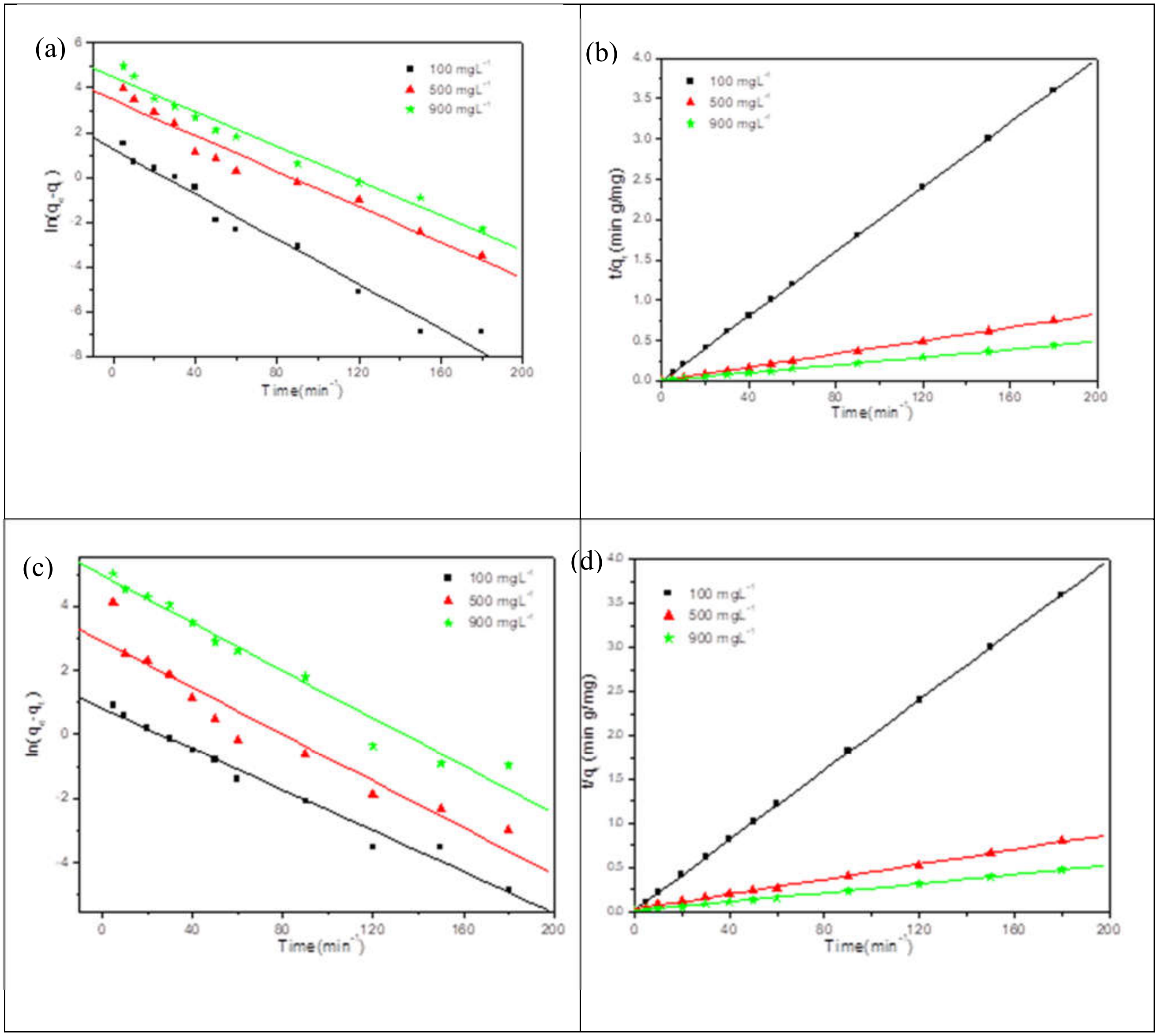

Figure S8. Pseudo-first-order and Pseudo-second-order kinetic models for the adsorption of Garamycin on MSG (a) and (b) respectively and Ampicillin on SG (c) and (d), respectively [Conditions: Dose of adsorbent: 2 mg/mL, Temperature: 25°C].

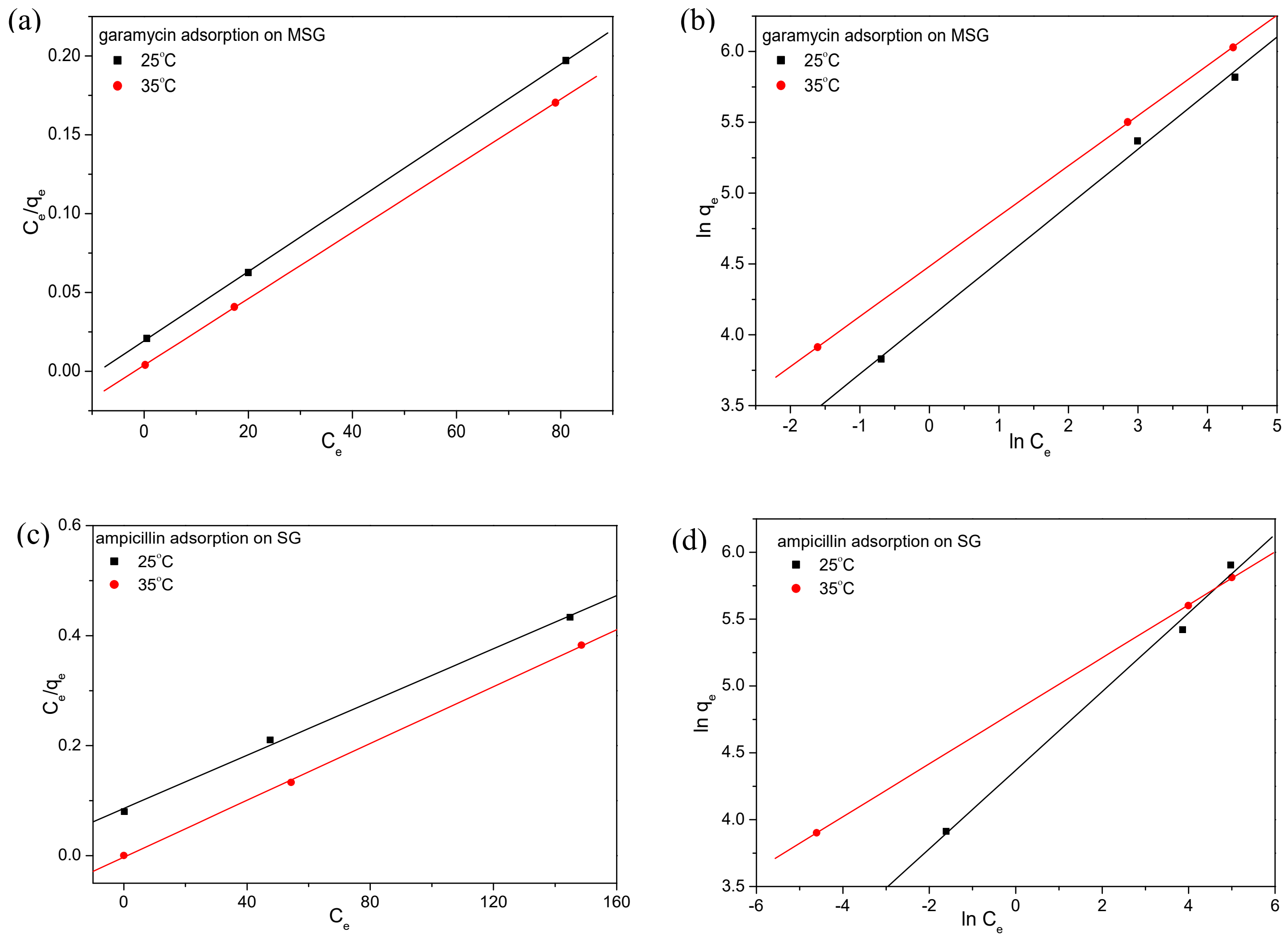

Figure S9. Langmuir and Freundlich isotherm models for the adsorption of Garamycin on MSG (a) and (b) respectively and Ampicillin on SG (c) and (d), respectively [Adsorbent dose: 2 mg/mL]

Table S1. Representative drug with their, antibiotics classification, IUPAC nomenclature, chemical structure, molecular formula, and molecular weight.

Representative drug	Antibiotics Classification	IUPAC Nomenclature	Chemical Structure	Chemical Formula	Molecular Weight g/mol
Garamycin	aminoglycosides	2-[4,6-diamino-3-[3-amino-6-[1-(methylamino)ethyl]oxan-2-yl]oxy-2-hydroxycyclohexyl]oxy-5-methyl-4-(methylamino)oxane-3,5-diol;sulfuric acid	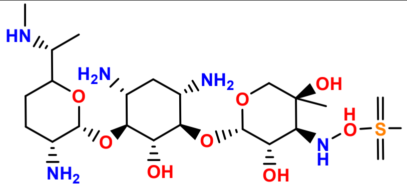	$C_{21}H_{45}N_5O_{11}S$	575.675
Ampicillin Sodium	aminopenicillin	sodium;(2S,5R,6R)-6-[[(2R)-2-amino-2-phenylacetyl]amino]-3,3-dimethyl-7-oxo-4-thia-1-azabicyclo[3.2.0]heptane-2-carboxylate	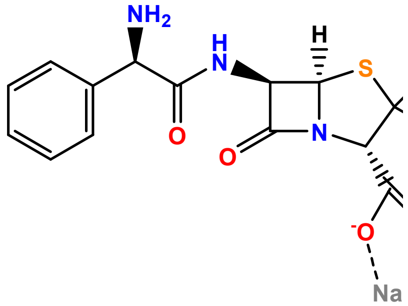	$C_{16}H_{18}N_3NaO_4S$	371.387

Table S2. The elemental composition and SO_3H contents of SG and MSG.

	SG	MSG
Elemental composition (wt.%)		
C	67.3	66.4
S	5.6	3.1
Fe	-	6.1
O	27.1	24.4
SO_3H contents (mmol g^{-1})	0.7	0.37

Table S3. Analysis of the main elements in the XPS survey spectra of MSG (At.% = atom%).

	MSG	
	**Peak B.E. (eV)**	**At. %**
C 1s	284.8	70.83
O 1s	533.0	26.58
S 2p	169.1	1.32
Fe 2p	711.9	1.27

Table S4. The Texture Feature (Surface Area, Pore Volume and Size) for G, SG, and MSG.

	HRGO	SG	MSG
BET surface area (m2 g^{-1})	702	475	607
total pore volume (cm^3 g^{-1})	0.386	0.288	0.469
average pore diameter (nm)	2.2	2.4	3.09

Table S5. pH values of the different solution.

	The Initial pH Aqueous Solution of Antibiotic	Antibiotic + SG	Antibiotic + MSG
Garamycin	5.6	4.8	5.6
Ampicillin	7.1	5.5	6.3

Table S6. Performance metrics of the prepared nanomaterials used for Garamycin and Ampicillin removal.

	Optimum pH	Initial Garamycin Concentration (mg L^{-1})	Initial Ampicillin Concentration (mg L^{-1})	Maximum Adsorption Capacity(mg g^{-1})	Maximum Removal Efficiency (%)	Distribution Coefficient (mg g^{-1} mM^{-1})
HRGO/ Garamycin	6	500	_	232.5	93	3824
HRGO/ Ampicillin	6	_	500	235	94	2909
MSG/Garamycin	5.6	500	_	240	96	6908
MSG/Ampicillin	6.3	_	500	155	62	303
SG/Garamycin	4.8	500	_	162.5	65	534
SG/Ampicillin	5.5	_	500	226	93.5	2583

Table S7. Central composite matrix of experimental and predicted values for Garamycin removal (%) using prepared MSG and Ampicillin removal (%) using prepared SG at solutions pH 5.5.

Trial	Time (X1; min)	AdsorbateInitial Concentration (X2;mg/L)	Adsorbent Dose (X3;mg)	Removal (%)			
				Measured		Predicted	
				Gara/MSG	Amp/SG	Gara/MSG	Amp/SG
**1**	60(0)	100(-1)	0.05(-1)	96.8	96	89.4	82.1
**2**	60(0)	900(1)	0.05 (-1)	89.4	82.1	91	83.9
**3**	60(0)	100(-1)	0.15 (1)	99.3	97.3	92.6	85.1
**4**	60(0)	900(1)	0.15 (1)	93.9	89.9	93.9	87.4
**5**	30(-1)	100(-1)	0.1 (0)	98	97	95.1	88.3
**6**	30(-1)	900(1)	0.1 (0)	91	83.9	96.8	89.9
**7**	90(1)	100(-1)	0.1 (0)	99	98.9	97	91.6
**8**	90(1)	900(1)	0.1 (0)	92.6	91.6	98	93.1
**9**	30(-1)	500(0)	0.05 (-1)	95.1	85.1	98.3	96.8
**10**	30(-1)	500(0)	0.15 (1)	98.3	88.3	98.7	97
**11**	90(1)	500(0)	0.05 (-1)	97	87.4	98.9	97.1
**12**	90(1)	500(0)	0.15 (1)	99.1	97.1	99.1	97.3
**13**	60(0)	500(0)	0.1(0)	98.7	93.1	99.3	98.9

Table S8.ANOVA analysis of the selected factors on the adsorption efficiency of MSG for garamycin in aqueous solution.

Regression Statistics					
**Multiple R**	0.998499				
R Square	0.996999				
Adjusted R Square	0.987997				
Standard Error	0.364005				
Observations	13				
ANOVA					
	df	*SS*	*MS*	*F*	*Significance F*
Regression	9	132.0717	14.67464	110.752	0.001268
Residual	3	0.3975	0.1325		
Total	12	132.4692			

Table S9.ANOVA analysis of the selected factors on the adsorption efficiency of SG for ampicillin in aqueous solution.

Regression Statistics					
Multiple R	0.995854				
R Square	0.991725				
Adjusted R Square	0.9669				
Standard Error	1.046821				
Observations	13				
ANOVA					
	df	*SS*	*MS*	*F*	*Significance F*
Regression	9	393.9956	43.77729	39.94885	0.005741
Residual	3	3.2875	1.095833		
Total	12	397.2831			

As illustrated in Tables S7, S8 and S9 of model validations, the agreement between the obtained and estimated removal efficiency showed that using response surface method to design the experiments can be considered as an effective choice in the optimization of process parameters besides its uses as an experimental design and statistical analysis.
